# The First Evidence of Lyme Neuroborreliosis in Southern Bosnia and Herzegovina

**DOI:** 10.1155/2014/231969

**Published:** 2014-12-15

**Authors:** Jurica Arapovic, Sinisa Skocibusic, Svjetlana Grgic, Jadranka Nikolic

**Affiliations:** ^1^Clinic for Infectious Diseases, University Hospital Mostar, Kralja Tvrtka bb, 88000 Mostar, Bosnia and Herzegovina; ^2^School of Medicine, University of Mostar, Bijeli Brijeg bb, 88000 Mostar, Bosnia and Herzegovina

## Abstract

Lyme borreliosis (LB) is caused by the spirochete *Borrelia burgdorferi*, which is transmitted to humans by ticks of the *Ixodes ricinus* complex. It is manifested by a variety of clinical symptoms and affects skin, joints, heart, and nervous system. Neurological manifestations are predictable and usually include meningoencephalitis, facial palsy, or radiculopathy. Recently, a dramatic rise in the number of diagnosed cases of LB has been observed on the global level. Here we show the first case of Lyme neuroborreliosis in southern Bosnia and Herzegovina, which was first presented by erythema chronicum migrans. Unfortunately, it was not recognized or well treated at the primary care medicine. After eight weeks, the patient experienced headache, right facial palsy, and lumbar radiculopathy. After the clinical examination, the neurologist suspected meningoencephalitis and the patient was directed to the Clinic for Infectious Disease of the University Hospital Mostar, where he was admitted. The successful antimicrobial treatment with the 21-day course of ceftriaxone was followed by normalization of neurological status, and then he was discharged from the hospital. This case report represents an alert to all physicians to be aware that LB is present in all parts of Bosnia and Herzegovina, as well as in the neighboring regions.

## 1. Introduction

Lyme disease or Lyme borreliosis (LB) is caused by the spirochete* Borrelia burgdorferi* sensu lato group with its human pathogenic species* B. burgdorferi* sensu stricto,* B. garinii*, and* B. afzelii*, which are transmitted by ticks of the species* Ixodes* [1, 2]. LB is manifested by a variety of clinical symptoms with occurrence of primary infection. It is a multiorganic disease affecting skin, joints, heart, and central nervous system, causing many neurological manifestations usually characterized by meningoencephalitis, facial palsy, or radiculopathy [2, 3]. LB can be divided into three stages. Of epidemiological importance is that the first stage of illness usually begins in the summer or spring, with presented erytheproteinorraahiama chronicum migrans at the site of the tick bite. The second stage of illness occurs within several days to weeks later, when the pathogen may spread to different parts of the body like the nervous system, heart, or joints. The third stage of illness may be developed up to several years after primary infection. This stage is characterized by persistent disease affecting the joints, nervous system, skin, and many other organs [1]. Of importance is that all these stages can be effectively treated with antimicrobial regiments [4].

Up to 80% of patients develop erythema chronicum migrans at the site of the tick bite [5]. However, in the absence of erythema chronicum migrans, physicians often have difficulties to diagnose LB [6]. The final diagnosis of LB is based on specific clinical symptoms that should be confirmed by serologic tests including ELISA, or more specifically, tests like Western blot [7].

From the epidemiological point of view, it is important to emphasize that, in many European countries and the United States, a dramatic increase of diagnosed patients with LB was observed [8]. Also, many previously unnoticed regions are now affected with this pathogen [3]. So far in our broader region, including Croatia and Serbia, it has been speculated that the occurrence of LB was generally restricted to Serbia, northern Croatia, and northern Bosnia and Herzegovina [9–12].

## 2. Case Description

Two months prior to admittance to the Clinic for Infectious Disease of the University Hospital Mostar, a 45-year old male was bitten by a tick in the area of right upper arm. He resided in a forest near the city of Mostar, which is located in southern Bosnia and Herzegovina. Several days later, a ring-shaped erythema appeared around the bitten area. The erythema was getting bigger and bigger, without affecting the other parts of the body. He visited a family medicine specialist at the Community Health Centre of Mostar, who treated him with cephalexin (1 g twice daily) for one week. Eight weeks later, the patient reported headache and neck pain. A day later, the pain went down to the regions of the back and legs. He again visited the Community Health Centre of Mostar, and the physician gave him nonsteroidal and opioid pain relievers. Despite strong analgesics, the symptoms did not decline. Then he visited the Clinic for Neurology of the University Hospital Mostar. A neurologist advised him to start the physical therapy and continue analgesic treatment. In the meantime, the patient was subjected to computed tomography (CT) of the lumbar region without any pathological finding. A few days later he presented with the right facial nerve palsy and interscapular pain, whereas the headache was still present. After the second neurological examination, the patient was directed to the Clinic for Infectious Disease of the University Hospital Mostar, where further treatment was initiated. All this time he had no fever. At admission to the hospital the patient had blood pressure of 140/80 mm Hg. All routine blood biochemical findings in sera were normal. Lumbar puncture was administrated and cerebrospinal fluid (CSF) analysis showed pleocytosis: 139 cells in 3 vision fields with 92% of lymphocytes, positive Pandy reaction, and elevated CSF total proteins 1.04 g/L (normal range is 0.15–0.45 g/L). An indirect immunofluorescence test for* B. burgdorferi *IgG antibodies in CSF was positive at ratio 1 : 256 (normal range < 1 : 2). The ELISA test for* B. burgdorferi *showed a high titer of IgM antibodies: 59.11 RU/mL, as well as IgG antibodies: 146.82 RU/mL (a result is considered positive when antibody titers exceed 22 RU/mL). Magnet resonance imaging (MRI) of the brain showed no abnormalities. Taking all together, these results suggested Lyme neuroborreliosis, which was manifested by facial palsy and radiculopathy. The twenty-one-day therapy with ceftriaxone (2 g once daily) was administered, supported by prednisone and vitamin B preparations. Also, the patient was daily rehabilitated by a physiotherapist and recovered during hospitalization. He was discharged with completely normal neurological status. He was followed up for 12 months, with completely normal neurological status and laboratory blood findings.

## 3. Discussion

In this paper we described the first case of Lyme neuroborreliosis in southern Bosnia and Herzegovina. A 45-year-old male patient presented with Bannwarth's syndrome [13]. This includes headache, facial palsy, and radiculopathy, as well as CSF lymphocytosis. LB is the vector-transmitted disease, which is sometimes difficult to diagnose, especially when neurological symptoms are dominant and final diagnosis may cost a lot [5, 14]. Of note is that cranial neuritis is presented by facial nerve palsy with up to 60% of all Lyme neuroborreliosis patients [1]. Here we presented for the first time the emergence of Lyme neuroborreliosis in southern Bosnia and Herzegovina. Several studies previously showed the presence of LB in the surrounding areas, mostly in northern Croatia and Serbia, but none of them reported the incidence of the disease in southern Bosnia and Herzegovina [9–12]. Thus, LB represents a growing public health threat in the world [3, 8]. Recent molecular and genetic studies have confirmed that* B. burgdorferi*, the spirochetal agent of LB, is one of the most complex bacteria known [15]. This should alert all physicians to take into account the presence of Lyme neuroborreliosis in southern Bosnia and Herzegovina as a new problem for epidemiologists and other physicians. Thus, they should put more effort to recognize LB on time and properly treat it.
